# Whole Genome Assembly of the Snout Otter Clam, *Lutraria rhynchaena*, Using Nanopore and Illumina Data, Benchmarked Against Bivalve Genome Assemblies

**DOI:** 10.3389/fgene.2019.01158

**Published:** 2019-11-20

**Authors:** Binh Thanh Thai, Yin Peng Lee, Han Ming Gan, Christopher M. Austin, Laurence J. Croft, Tuan Anh Trieu, Mun Hua Tan

**Affiliations:** ^1^Fisheries and Technical Economical College, Tu Son, Vietnam; ^2^Centre of Integrative Ecology, School of Life and Environmental Sciences, Deakin University, Geelong, VIC, Australia; ^3^Deakin Genomics Centre, Deakin University, Geelong, VIC, Australia; ^4^Faculty of Biology, Ha Noi National University of Education, Ha Noi, Vietnam; ^5^Science and Technique Department, Hung Vuong University, Viet Tri, Vietnam

**Keywords:** snout otter clam, genome, hybrid assembly, aquaculture, Illumina, Oxford Nanopore

## Introduction

Production of cultured bivalve molluscs was 17.1 million tons in 2016 accounting for 21.4% of global aquaculture production (FAO, 2018). The lack of genomic resources coupled with limited understanding of the molecular basis of gene expression and phenotypic variation have limited advances in aquaculture-based productivity of marine bivalves. Understanding the molecular basis of phenotypic variation and gene function is therefore important for selective breeding programs for traits such as increased growth and disease resistance. Similarly, whole genome assemblies support GWAS studies to identify trait-specific loci and for genomic-based selective breeding. To this end, whole-genome sequencing has been conducted on several commercial bivalve species, including the edible oysters *Crassostrea virginica* (Gómez-Chiarri et al., 2015), *Crassostrea gigas* (Gerdol et al., 2015), pearl oysters (Takeuchi et al., 2012) and clams (Mun et al., 2017). However, in general, genomic data for bivalve molluscs, which includes a taxonomically diverse group of species, are sparse (Takeuchi et al., 2012; Zhang et al., 2012; Murgarella et al., 2016; Du et al., 2017; Li et al., 2017; Mun et al., 2017; Sun et al., 2017; Uliano-Silva et al., 2017; Wang et al., 2017; Li et al., 2018; Powell et al., 2018; Renaut et al., 2018; Bai et al., 2019). In this study we present the first genomic resources for a species of clam from the superfamily Mactroidea and for a Vietnamese shellfish species and generate a draft reference genome to form the basis of on-going selective breeding studies. This study also demonstrates the efficacy of using Oxford Nanopore Technology (ONT) reads to scaffold bivalve genome assemblies and shows the value of these relatively inexpensive long reads for spanning large repetitive regions and overcoming complex assembly issues caused by high heterozygosity, which typically confounds short read only assemblies. The quality of our assembly is also benchmarked against other bivalve genome assemblies and we present an initial phylogenomic analysis for the class Bivalvia, which illustrates the value and potential of the increasing number of high quality genomic data sets for phylogenetics.

## Materials and Methods

### Extraction, Library Preparation, and Sequencing

Muscle tissue was collected from a sample obtained from a snout otter clam farm in Van Dong district, Quang Ninh province in Vietnam. For short read sequencing of the clam genome, genomic DNA (gDNA) was extracted according to Sokolov’s improved method (Sokolov, 2000). Two sequencing libraries were prepared. These include a PCR-free library prepared using NuGen Celero DNA-Seq Library Preparation Kit (Tecan Genomics, San Carlos, CA) according to manufacturer’s instruction and a PCR-based library prepared with NEBNext Ultra DNA Library Preparation Kit (New England Biolabs, Ipwich, MA). For transcriptomic data, RNA was extracted from digestive gland tissue using the Zymo Quick-RNA Miniprep kit, followed by RNA library constructed using the Nugen Universal Plus mRNA-Seq Kit (Tecan Genomics, San Carlos, CA) according to manufacturer’s instruction. Both DNA and RNA libraries were sequenced on an Illumina NovaSeq6000 platform at the Deakin Genomics Centre with 2 × 150 bp configuration. For Nanopore long read sequencing, gDNA was extracted from the muscle tissue of the same individual using the column-based Zymo Quick DNA miniprep plus. Approximately 1 µg of the purified gDNA was used as the input for library preparation using the LSK109 kit followed by sequencing on a FLO-MIN106 revD SpotON R9.4 Flow Cell for 48 h.

### Genome Size Estimation

Short reads generated by the Illumina NovaSeq SGS platform were preprocessed for genome size estimation. The fastp v0.19.4 tool (Chen et al., 2018) was used to trim polyG tails from the 3’ end of short reads (–*poly_g_min_len 1*), followed by adapter and quality trimming with Trimmomatic v0.36 (Bolger et al., 2014) (*ILLUMINACLIP:2:30:10, AVGQUAL:20*). Reads of mitochondrial origin were removed *via* alignment against the *Lutraria rhynchaena* mitogenome (Gan et al., 2016) using bowtie2 v2.3.3.1 (default parameters) (Langmead and Salzberg, 2012). Jellyfish v2.2.6 (Marçais and Kingsford, 2011) was used to count the frequency of 19-, 21- and 25-mers in the preprocessed reads and finally, these k-mer histograms were uploaded to the GenomeScope webserver (*max kmer coverage* disabled) (Vurture et al., 2017) for an estimation of the *L. rhynchaena* haploid genome size.

### *De Novo* Hybrid Assembly of the Snout Otter Clam Genome

Long reads generated by the ONT MinION sequencing device were basecalled and trimmed (adapter and quality) with Guppy v3.0.3 (*high accuracy mode, min_qscore 7*), which is available *via* the ONT community site (https://community.nanoporetech.com). A hybrid *de novo* assembly approach was performed with the MaSuRCA v3.2.8 assembler (*USE_LINKING_MATES = 0*, *cgwErrorRate = 0.15*, *KMER_COUNT_THRESHOLD = 1*) (Zimin et al., 2013), using the previously trimmed long Nanopore reads and polyG-trimmed short Illumina reads as input. The MaSuRCA assembler first reduces high coverage Illumina reads into longer super-reads, then aligns these to long Nanopore reads to build even longer mega-reads (Zimin et al., 2017). These are then assembled by the CABOG assembler within MaSuRCA. Additionally, this version of MaSuRCA uses the high coverage of the longer more error prone Nanopore reads to build consensus sequences for regions not captured by short accurate Illumina reads. As a result, this approach combines the benefits from the accuracy of Illumina reads and the length of Nanopore reads. Completeness of the assembly was assessed with BUSCO v3.0.2 (Simão et al., 2015), using single-copy orthologs from the Metazoan dataset (metazoa_odb9).

As bivalve genomes have been previously shown to be highly heterozygous (Zhang et al., 2012; Wang et al., 2017; Powell et al., 2018), an observation also apparent from the bimodal distribution of k-mer profiles obtained from the GenomeScope analysis in this study (**Data Sheet 1**), it was necessary to optimize the assembly by removing haplotigs or duplications in the haploid representation of this genome. This was achieved by aligning the long reads back to the assembly with minimap2 v2.15 (-*x map_ont*, –*secondary = no*) (Li, 2018) and passing the alignment through the Purge Haplotigs pipeline v1.0.4 (-*l 5*, -*m 20*, -*h 150*) (Roach et al., 2018) to remove artifactual scaffolds.

### Estimation of Heterozygosity and Repeat Content

Presence of single nucleotide polymorphisms (SNP) in the snout otter clam genome was estimated by aligning short reads to the final curated assembly with bowtie2 v2.3.3.1 (Chen et al., 2018) and called with the *mpileup* (–*max-depth 200*) and *call* (-*mv*) commands within bcftools v1.9 (Li, 2011), retaining high quality calls with sufficient read depth (*QUAL* ≥ *20*, DP ≥ 10, AF ≥ 0.25). Repeat families within the final assembly were identified *de novo* with RepeatModeler v1.0.11 (default parameters) (Smit and Hubley, 2019), which uses both RECON v1.08 (Bao and Eddy, 2002) and RepeatScout v1.0.5 (Price et al., 2005) to search for repeats within a given assembly. From this, a set of consensus sequences for each identified family was then used to mask repeats within the assembly with RepeatMasker v4.0.9 (-*e rmblast*) (Smit et al., 2018).

### Benchmarking Against Other Bivalve Genomes

The assembly sizes, scaffold N_50_ lengths and other characteristics of the *L. rhynchaena* assembly were compared with 13 other published bivalve genomes representing six orders and eight families. These comprise: *Argopecten purpuratus* (Li et al., 2018), *Bathymodiolus platifrons* (Sun et al., 2017), *Chlamys farreri* (Li et al., 2017), *C. gigas* (Zhang et al., 2012), *Limnoperna fortunei* (Uliano-Silva et al., 2017), *Modiolus philippinarum* (Sun et al., 2017), *Mytillus galloprovincialis* (Murgarella et al., 2016), *Patinopecten yessoensis* (Wang et al., 2017), *Pinctada fucata* (Takeuchi et al., 2012; Du et al., 2017), *Ruditapes philippinarum* (Mun et al., 2017), *Saccostrea glomerata* (Powell et al., 2018), *Scapharca broughtonii* (Bai et al., 2019) and *Venustaconcha ellipsiformis* (Renaut et al., 2018). Assessments of repeat content and BUSCO completeness (metazoa_odb9) were repeated for these 13 bivalve genomes as described above.

### Transcriptome Assembly

One RNA-seq library was sequenced and a transcriptome was generated to assist in gene prediction. Paired-end, strand-specific RNA reads were polyG-, adapter- and quality-trimmed with the same tools and parameters as outlined for genomic reads. The resulting reads were assembled *de novo* with the Trinity v2.8.5 assembler (–*SS_lib_type FR*) (Grabherr et al., 2011). Short reads were subsequently mapped back to the assembly with bowtie2 v2.3.3.1 (Langmead and Salzberg, 2012). Additionally, GMAP v2019-06-10 (default parameters) (Wu and Watanabe, 2005) was used to align the assembled transcriptome to the current genome assembly.

### Gene Prediction and Annotation

Gene prediction was carried out with the MAKER v2.31.10 annotation pipeline (Holt and Yandell, 2011). Providing this pipeline with the previously identified repeat families, the assembled transcript sequences and a set of protein sequences from the 13 published bivalve genomes (**Data Sheet 2**) (Takeuchi et al., 2012; Zhang et al., 2012; Murgarella et al., 2016; Du et al., 2017; Li et al., 2017; Mun et al., 2017; Sun et al., 2017; Uliano-Silva et al., 2017; Wang et al., 2017; Li et al., 2018; Powell et al., 2018; Renaut et al., 2018; Bai et al., 2019), MAKER identifies repeats and subsequently aligns transcripts and proteins to the genome to produce initial gene models and sequences in its first iteration (*est2genome = 1*, *protein2genome = 1*). These gene models were used to train two *ab initio* gene prediction softwares AUGUSTUS (using BUSCO with –*long* and metazoa_odb9) (Stanke et al., 2006) and SNAP (-*categorize 1000*, -*export 1000*) (Korf, 2004), followed by a second iteration of MAKER incorporating gene models generated from *ab initio* predictors. Gene models were again retrained with a second iteration of AUGUSTUS and SNAP before MAKER was repeated for a third iteration.

Sequences with Annotation Edit Distance (AED) values ≤0.5 were retained. AED values are evidence-based (Eilbeck et al., 2009), whereby a small value suggests a lesser degree of difference between the predicted gene and the protein and/or transcript evidence used during prediction. These protein sequences were further aligned against proteins in UniProtKB (Swiss-Prot and TrEMBL) (Consortium, 2018) for homology using DIAMOND v0.9.24.125 (–*max_target_seqs 1*, –*evalue 1e* *^−10^*) (Buchfink et al., 2014) and searched for protein domains and signatures with InterProScan v5.36-75.0 (-*t p*) (Jones et al., 2014).

### Maximum Likelihood and Bayesian Phylogenetic Analyses

BUSCO-predicted protein-coding genes were used to construct a multi-gene supermatrix in order to infer the phylogenetic position of *L. rhynchaena* in relation to bivalve species for which published genomes are available (**Data Sheet 2**), with the exception of *M. galloprovincialis.* This was excluded from this analysis since its assembly reported substantially lower BUSCO completeness. The owl limpet (*Lottia gigantea*), two-spot octopus (*Octopus bimaculoides*) and fruit fly (*Drosophila melanogaster*) were included as outgroup species. Each orthologous group was aligned with MAFFT v7.394 (default parameters) (Katoh and Standley, 2013) and trimmed with Gblocks v0.91b (default parameters) (Castresana, 2000), allowing no gaps in the alignments. Only orthologous protein groups with 100% representation (i.e. contains proteins from all 13 bivalves and three outgroups) were incorporated to form the final supermatrix. This supermatrix (22,668 amino acids, 129 orthologous groups), partitioned by proteins (-*spp*), was supplied to IQ-TREE v1.5.5 (-*bb 1000*, -*alrt 1000*, -*m TESTMERGE*) (Nguyen et al., 2014) for model testing and to infer phylogenetic relationships using maximum likelihood (ML) with nodal support assessed using Ultrafast Bootstrap support (UFBoot) (Minh et al., 2013) and SH-aLRT (Guindon et al., 2010) values. The same supermatrix was supplied to ExaBayes v1.5 (Aberer et al., 2014) to infer a Bayesian tree (BI) from four independent runs of two million generations. After discarding 25% of initial samples as burn-in, convergence was determined when the average standard deviation of split frequencies (*asdsf*) value fell below 1%.

## Results and Discussion

### Genome Size and Sequencing Coverage

This study generated a large volume of genomic data made up of 122 Gbp of short paired-end reads (150 bp) and 14 Gbp of long reads (average: 4,960 bp, longest: 58,804 bp, shortest: 200 bp) (**Data Sheet 3**). In addition, 34 Gbp of transcriptomic reads were generated to facilitate gene prediction. Raw Illumina and Nanopore reads generated in this study are available in the Sequence Read Archive (SRP201027) under BioProject PRJNA548223.

The snout otter clam genome size was estimated to range from 545 to 547 Mbp based on 19-, 21- and 25-mers as summarised in **Data Sheet 1**, with histograms provided in **Data Sheet 4**. Based on these estimates this study generated short and long read data with sequencing depths of over 200× and 25×, respectively. The haploid genome size for the snout otter clam is at the lower end of a large range of estimated genome sizes for the species of the family Mactridae and also within the phylum Bivalvia more generally (**Data Sheet 5**).

### Genome and Genes of the Snout Otter Clam

The snout otter genome assembly has used the largest volume of ONT data generated for a bivalve species to date and joins the blood clam (*S. broughtonii*) (Bai et al., 2019) as the only other bivalve genome assembly to incorporate the use of long Nanopore reads and has generated the largest volume of Nanopore data for a bivalve species to date. As the first study to use a combination of only Illumina paired-end reads and Nanopore long reads, it also demonstrates the efficacy of the addition of Nanopore reads as a cost-effective option to achieve a high quality and contiguous genome assembly in combination with Illumina reads. This hybrid assembly approach has generated one of the best bivalve draft genomes currently available consisting of only 1,502 scaffolds (N_50_ length: 1.84 Mbp) and a total assembly size of 586.5 Mbp (**Table 1**), slightly exceeding the kmer-based genome size estimates. The assembly displayed a high overall completeness of 95.9%. However, 2.9% of the metazoan BUSCOs detected in this assessment occurred in duplicates within the assembly, likely to be a result of the elevated heterozygosity of the genome, which is not uncommon for marine invertebrate species. These duplicated regions can cause issues in downstream analyses such as variant discovery and therefore were removed from the assembly before its use for other applications. This then produced a final curated assembly of 544 Mbp contained in 622 scaffolds (N_50_ length: 2.14 Mbp) (**Table 1**), to which 95.6% of Illumina short reads were successfully aligned using bowtie2 v2.3.3.1 (Langmead and Salzberg, 2012). BUSCO reports a similar overall completeness of 95.8% but with 1.5% BUSCOs detected in duplicates, half of that reported prior to the haplotig purging strategy. This Whole Genome Shotgun project has been deposited at DDBJ/EMBL/GenBank under the accession VIBL00000000 under BioProject PRJNA548223.

**Table 1:** Summary of assembly and annotation of the snout otter clam genome.

	*Lutraria rhynchaena*	
	MaSuRCA	MaSuRCA + Purge Haplotigs
**Genome Size Estimation**		
Based on 21-mer counts	546,627,519 bp	
**Genome Assembly**		
Assembly size	586,528,440 bp	543,903,147 bp
Number of scaffolds	1,502	622
Scaffold N50 length	1,841,940 bp	2,143,760 bp
Average scaffold length	390,498 bp	874,442 bp
Longest scaffold	13,022,122 bp	13,022,122 bp
Shortest scaffold	1,560 bp	2,218 bp
**Genome Completeness (Metazoan BUSCOs: 978 total)**		
Complete BUSCOs	95.9%	95.8%
Complete and single BUSCOs	93.0%	94.3%
Complete and duplicated BUSCOs	2.9%	1.5%
Fragmented BUSCOs	0.3%	0.3%
Missing BUSCOs	3.8%	3.9%
**Genome Annotation**		
Number of predicted PCGs	–	26,380
Number of annotated PCGs	–	23,701
Hits to UniProtKB	–	18,595
Hits to protein domains/signatures	–	23,114
Average length of PCGs	–	379.61 aa
Longest PCG	–	9,456 aa

Variant calling analysis detected a total 4,903,576 SNPs, which translates into a heterozygosity estimate of 0.90%. In contrast, heterozygosity was estimated at 1.60% using the kmer-based approach with GenomeScope (**Data Sheet 1**). Since variant calling methods are typically more conservative than kmer-based ones, the former approach may have excluded some true positives and, thereby leading to an underestimation of genetic variation. Nevertheless, the heterozygosity rates estimated by both methods (0.90 and 1.60%) is within the range of values that have been reported for other bivalves (0.51 to 2.02%). Repeat modelling and masking of the genome masked 29.4% of the *L. rhynchaena* assembly.

This study also generated 34.6 Gbp of short paired-end, strand specific RNA reads. The resultant transcriptome assembly produced a set of 295,234 transcripts containing 83.9% complete BUSCO genes. A total of 96.4% of these RNA reads were mapped back to this set of assembled transcripts and of these, 79% were aligned to the genome in a splice-aware manner. Both alignment rates indicate that the quality of the set of transcripts is sufficient to improve the gene prediction. Based on hints and evidence from the clam transcripts and protein sequences from other bivalves, gene prediction from the assembled genome resulted in a final set of 26,380 protein-coding genes (AED ≤0.5), 89.8% of which were functionally annotated (i.e. a protein would have at least one associated functional annotation) (Table 1). The assembled transcriptome, predicted protein-coding genes and annotation information are available as **Data Sheet 6** and **Data Sheet 7**.

### Comparison Against Other Bivalves

**Figure 1:**
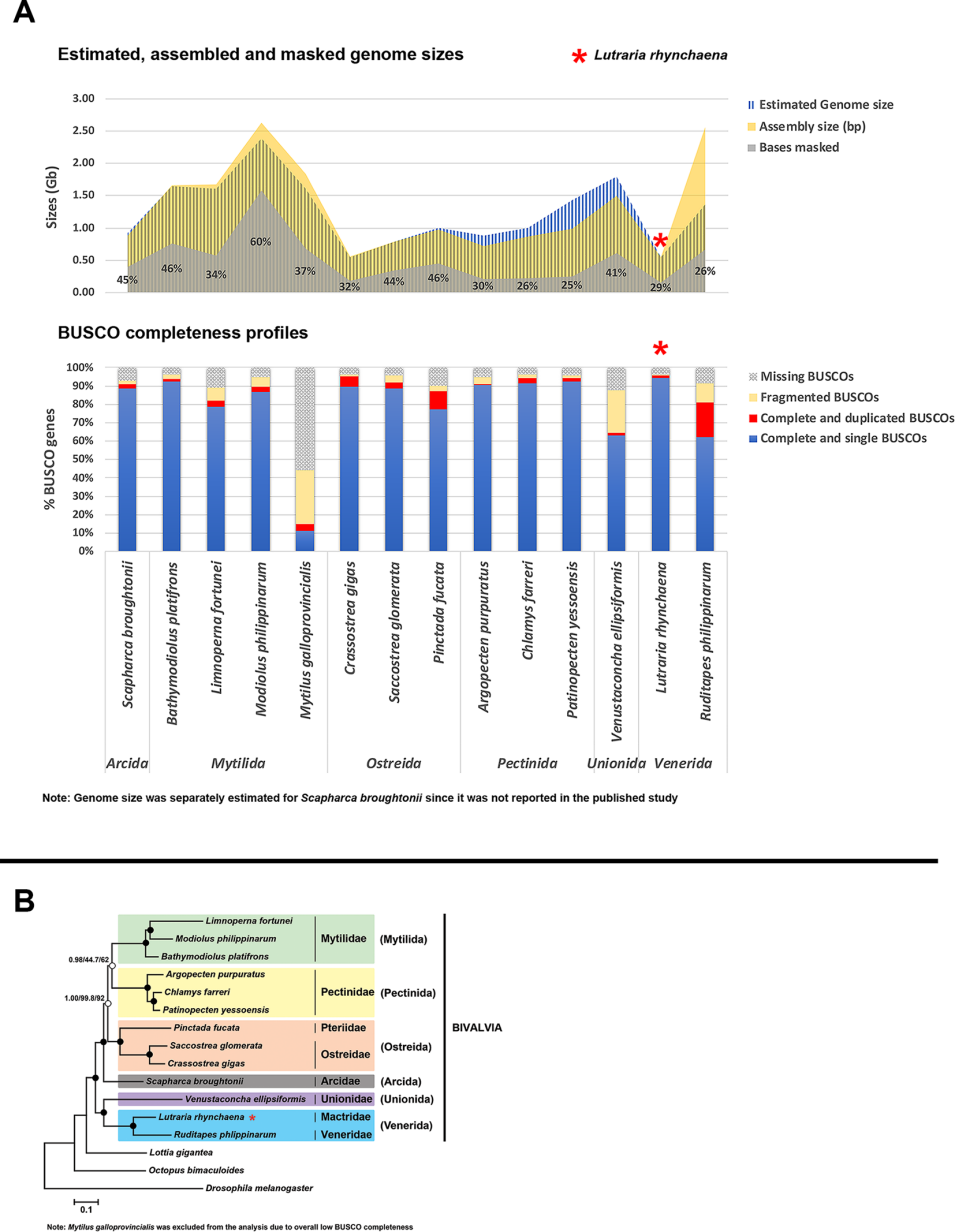
Comparison of *Lutraria rhynchaena* against other published bivalve assemblies. **(A)** Comparison of BUSCO completeness, estimated vs assembled genome sizes and repeat content. **(B)** Evolutionary relationships among bivalve species inferred from maximum likelihood (ML) and Bayesian (BI) methods. Rooted with *Drosophila melanogaster*, the shown BI topology was inferred from a supermatrix of 22,668 amino acid characters was constructed from the alignments of 129 orthologous protein groups. Closed circles indicate maximum nodal support values (BPP/SH-aLRT/UFBoot).

A summary of assembly sizes, scaffold N_50_ lengths, sequencing technologies and other information for all 13 assemblies is available in **Data Sheet 2** , with a comparison of repeat content, genome and assembly sizes and BUSCO completeness for all 14 bivalve genomes is visualised in **Figure 1**. The assembly for *M. galloprovincialis* has a high level of missing BUSCO genes (14.8% complete, 29.6% fragmented, 55.6% missing) whereas the *V. ellipsiformis* assembly contained relatively more fragmented BUSCO genes (22.8%). This potentially points to limitations of the data types used, since the former was assembled with only short Illumina paired-end reads, while the latter had both paired-end (PE) and mate-pair (MP) reads but was assisted by only 0.3× of PacBio long reads. While three other assemblies also had only PE and MP reads, most of the remaining assemblies employed the use of other scaffolding strategies including a greater volume of long PacBio reads, fosmid and bacterial artificial chromosome (BAC) libraries (see **Data Sheet 2** for details). Furthermore, there is a large discrepancy in estimated genome size and assembly size for *R. philippinarum* (**Figure 1**), where the reported assembly is almost twice the size of the expected genome size. For this assembly, a high proportion of duplicated BUSCO genes was also detected (19.1%), highlighting the potential importance of haplotig purging within bivalve assemblies to remove paralogous scaffolds. Nevertheless, this step should be executed with caution as there is also the potential to “over-purge” and exclude actual parts of the genome.

### Phylogeny of Bivalvia Based on Nuclear Loci

Phylogenies inferred by both methods showed strong UFBoot, SH-aLRT and Bayesian posterior probability (BPP) support for most nodes, the exception being the sister relationship between the bivalve orders Mytilida and Pectinida (**Figure 1**). The snout otter clam, *L. rhynchaena*, is the only representative for Mactridae and forms a sister relationship with *R. philippinarum* from the Veneridae, consistent with their placement in the order Venerida. This initial phylogenomic analysis of the Bivalvia contains species from the three subclasses Imparidentia, Palaeoheterodonta, and Pteriomorpha. All bivalve species are placed within groups consistent with their taxonomic classification at the family and ordinal levels and observed relationships among these three subclasses are consistent with findings from recent studies based on Sanger and transcriptome sequencing (Kocot et al., 2011; González Vanessa et al., 2015; Combosch et al., 2017; Lemer et al., 2019). While these studies include greater taxon sampling from a range of bivalve subclasses, the data matrices used to infer phylogeny are often gappy with a higher level of missing data (∼16 to 75%). In contrast, our study successfully used information from a substantial number of genes (> 100) across across a wide density of bivalve taxa with minimal gaps. Nevertheless, our approach to phylogenomics is limited for the time being by the scarcity in genome level resources for bivalve species generally and specifically in relation to missing representatives from important bivalve subclasses including Protobranchia, Archiheterodonta and Anomalodesmata.

This initial phylogenomic analysis for the Bivalvia demonstrates the value and potential of this approach as a greater number of high quality genomic data sets become available that can be used for phylogenetic studies, especially for the resolution of basal relationships in what has been a challenging group (Plazzi et al., 2011; Sharma et al., 2012) Multiple sequence alignment and the resulting Newick tree from our analyses are available as **Data Sheet 8.**

## Conclusion

We present the first draft genome of the snout otter clam (*L. rhynchaena*) based on relatively large volumes of short and long genomic reads from Illumina and ONT platforms, respectively, providing sufficient sequencing depth to facilitate the generation of one of the best quality genome assemblies for a bivalve mollusk. The use of long Nanopore reads in a hybrid assembly, presents an effective yet economical approach to achieving a good quality assembly and highlights the importance of long reads in spanning large repeat regions and resolving other complex regions that arises for species with high levels of heterozygosity. This highly contiguous and complete assembly makes this draft genome an important and valuable resource to support ongoing genomic and molecular-based breeding studies for aquaculture. In addition, a transcriptomic data set was generated and assembled to support more refined gene prediction, further adding to the currently scarce transcriptomic resources available for the Mactridae family. Ultimately, we expect results of this study to be used as valuable genomic references for a range of genetic, genomic, phylogenetic and population studies of the snout otter clam and other bivalve species.

## Data Availability Statement

The datasets generated for this study can be found in the NCBI BioProject PRJNA548223 (WGS accession: VIBL00000000, SRA accession: SRP201027).

## Author Contributions

BT and CA conceived the project. TT collected the samples. YL and HG performed the sequencing. MT analyzed the data. MT and CA wrote the manuscript. MT, CA, BT, YL, HG, TT and LC read and reviewed the manuscript.

## Funding

This study was funded through a grant to Binh Thanh Thai from the Government of Vietnam through its Aquaculture Biotechnology programs (01/2017/HÐ-TS-CNSH) and the Deakin Genomics Centre, Deakin University, Australia.

## Conflict of Interest

The authors declare that the research was conducted in the absence of any commercial or financial relationships that could be construed as a potential conflict of interest.

## Supplementary Material

Data Sheet 1 Estimation of genome size, repeat content and heterozygosity by GenomeScope.

Data Sheet 2 Comparisons of published bivalve genome assemblies.

Data Sheet 3 Sequence methods and library kits used in the hybrid genome assembly.

Data Sheet 4 19-, 21-, 25-mer histograms generated by Jellyfish.

Data Sheet 5 Distribution of genome sizes of bivalve species.

Data Sheet 6 Snout otter clam transcriptome.

Data Sheet 7 Protein sequences predicted from the snout otter clam genome and associated annotation.

Data Sheet 8 Supermatrix and phylogenetic tree for the bivalve clade.
