# Supplementary material for: Whole Genome Assembly of the Snout Otter Clam, Lutraria rhynchaena, Using Nanopore and Illumina Data, Benchmarked Against Bivalve Genome Assemblies
Source: Front Genet. 2019 Nov 20;10:1158. doi: 10.3389/fgene.2019.01158 (PMC6880199; doi:10.3389/fgene.2019.01158)

Supplementary. Estimation of genome size, repeat content and heterozygosity by GenomeScope, based on 19-, 21- and 25-mers in Illumina NovaSeq sequence reads (*max kmer coverage* disabled).

<u>19-mer</u>

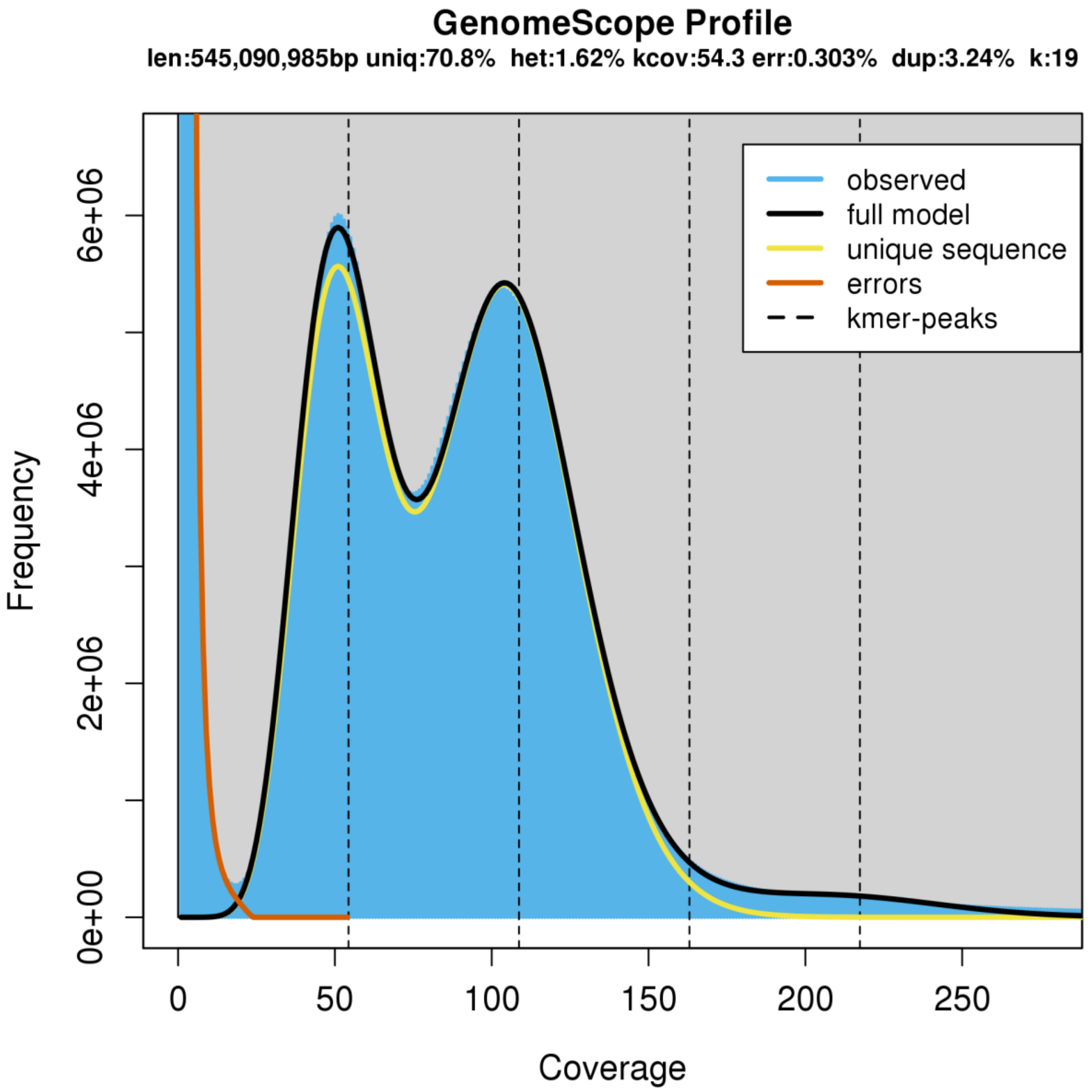

Supplementary. Estimation of genome size, repeat content and heterozygosity by GenomeScope, based on 19-, 21- and 25-mers in Illumina NovaSeq sequence reads (*max kmer coverage* disabled).

21-mer

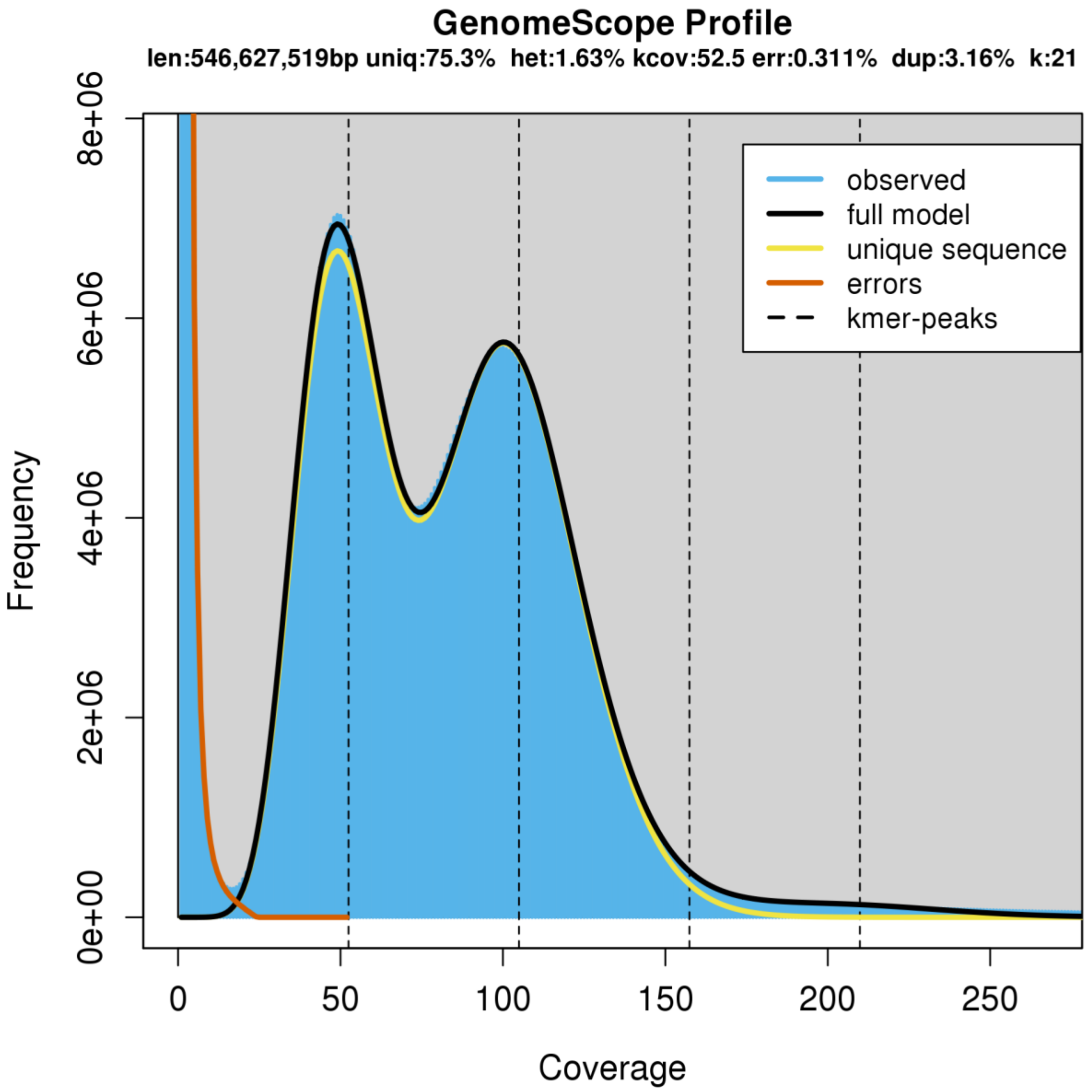

Supplementary. Estimation of genome size, repeat content and heterozygosity by GenomeScope, based on 19-, 21- and 25-mers in Illumina NovaSeq sequence reads (*max kmer coverage* disabled).

25-mer

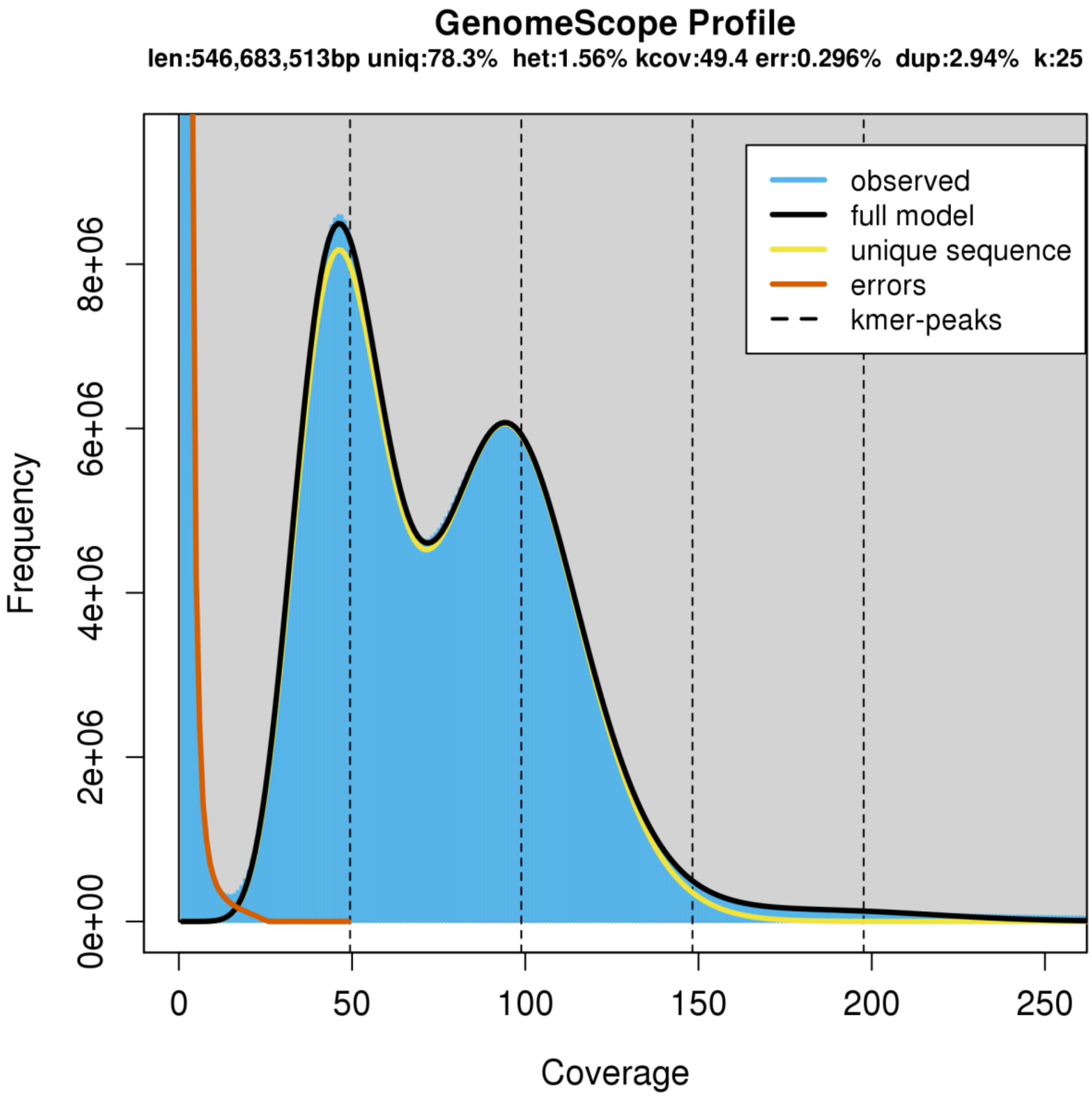

Supplement: Data Sheet 1 — Estimation of genome size, repeat content and heterozygosity by GenomeScope. [file DataSheet_1.pdf]
