# Supplementary material for: Whole Genome Assembly of the Snout Otter Clam, Lutraria rhynchaena, Using Nanopore and Illumina Data, Benchmarked Against Bivalve Genome Assemblies
Source: Front Genet. 2019 Nov 20;10:1158. doi: 10.3389/fgene.2019.01158 (PMC6880199; doi:10.3389/fgene.2019.01158)
Supplement: Data Sheet 2 — Comparisons of published bivalve genome assemblies. [file DataSheet_2.pdf]

Comparisons of published bivalve genome assemblies.

Order	Family	Species	Assembly size (bp)	Number of scaffolds	Scaffold N_{50} length (bp)	Longest scaffold (bp)	Shortest scaffold (bp) †	Sequencing Technology	Published studies
Arcida	Arcidae	*Scapharca broughtonii*	884,566,040	1,026	44,995,656	55,667,740	2,698	PE, PB, ONT, Hi-C	Bai et al. (2019)
Mytilida	Mytilidae	*Bathymodiolus platifrons*	1,658,191,953	65,662	343,341	2,790,175	292	PE, MP	Sun et al. (2017)
		Limnoperna fortunei	1,673,223,206	20,580	309,123	2,720,304	558	PE, MP, PB	Uliano-Silva et al. (2017)
		Modiolus philippinarum	2,629,556,424	74,573	100,161	715,382	205	PE, MP	Sun et al. (2017)
		Mytilus galloprovincialis	1,836,276,477	1,188,859	3,074	67,529	200	PE	Murgarella et al. (2016)
Ostreida	Ostreidae	*Crassostrea gigas*	557,717,710	7,658	401,685	1,964,558	200	PE, MP, Fos	Zhang et al. (2012)
		Saccostrea glomerata	788,100,799	10,101	804,232	7,146,926	504	PE, MP, Hi-C	Powell et al. (2018)
	Pteriidae	*Pinctada fucata*	972,756,244	1,562	59,032,463	104,615,532	1,737	PE, MP, BAC	Du et al. (2017)
Pectinida	Pectinidae	*Argopecten purpuratus*	724,780,576	89,727	1,022,003	11,125,544	200	PE, MP, PB, 10X	Li et al. (2018)
		Chlamys farreri	874,029,444	92,847	711,029	6,573,047	200	PE, MP, PB	Li et al. (2017)
		Patinopecten yessoensis	987,568,220	82,658	803,631	7,498,238	200	PE, MP	Wang et al. (2017)
Unionida	Unionidae	*Venustaconcha ellipsiformis*	1,590,012,607	371,427	6,657	313,274	1001	PE, MP, PB	Renaut et al. (2018)
Venerida	Mactridae	*Lutraria rhynchaena*	543,903,147	622	2,143,760	13,022,122	2,218	PE, ONT	**This study**
	Veneridae	*Ruditapes philippinarum*	2,561,070,351	223,851	48,447	572,939	500	PE, MP, TSLR	Mun et al. (2017)

† A length cutoff of 200 bp was applied to all assemblies that included smaller scaffolds.

Sequencing Technology abbreviations:

PE: Illumina Paired-end short reads
MP: Illumina Mate-pair short reads
PB: Pacific Biosciences long reads
ONT: Oxford Nanopore Technology long reads
Hi-C: Hi-C scaffolding (chromosome construction)
TSLR: Illumina TruSeq Synthetic Long Read
Fos: Fosmid-pooling strategy
BAC: BAC sequencing
10X: 10X Genomics library
