# Supplementary material for: Whole Genome Assembly of the Snout Otter Clam, Lutraria rhynchaena, Using Nanopore and Illumina Data, Benchmarked Against Bivalve Genome Assemblies
Source: Front Genet. 2019 Nov 20;10:1158. doi: 10.3389/fgene.2019.01158 (PMC6880199; doi:10.3389/fgene.2019.01158)
Supplement: Data Sheet 3 — Sequence methods and library kits used in the hybrid genome assembly. [file DataSheet_3.pdf]

Sequence methods and library kits used in the hybrid assembly of snout otter clam (*L. rhynchaena*) genome

	Platform	Read length	Number of Reads	Total Bases	Estimated genome coverage
Genomic	Illumina (NovaSeq) - PCR-free	2 × 150bp	367,711,652	55.16 Gbp	100×
	Illumina (NovaSeq) - PCR	2 × 150bp	445,111,088	66.77 Gbp	121×
	ONT (MinION)	variable	2,830,557	14.04 Gbp	25×
Transcriptomic	Illumina (NovaSeq)	2 × 150 bp	230,852,564	34.63 Gbp	N/A
