# Supplementary figures and images for: Whole Genome Assembly of the Snout Otter Clam, Lutraria rhynchaena, Using Nanopore and Illumina Data, Benchmarked Against Bivalve Genome Assemblies
Source: Front Genet. 2019 Nov 20;10:1158. doi: 10.3389/fgene.2019.01158 (PMC6880199; doi:10.3389/fgene.2019.01158)

## Distribution of genome sizes of bivalve species reported in the Animal Genome Size Database.

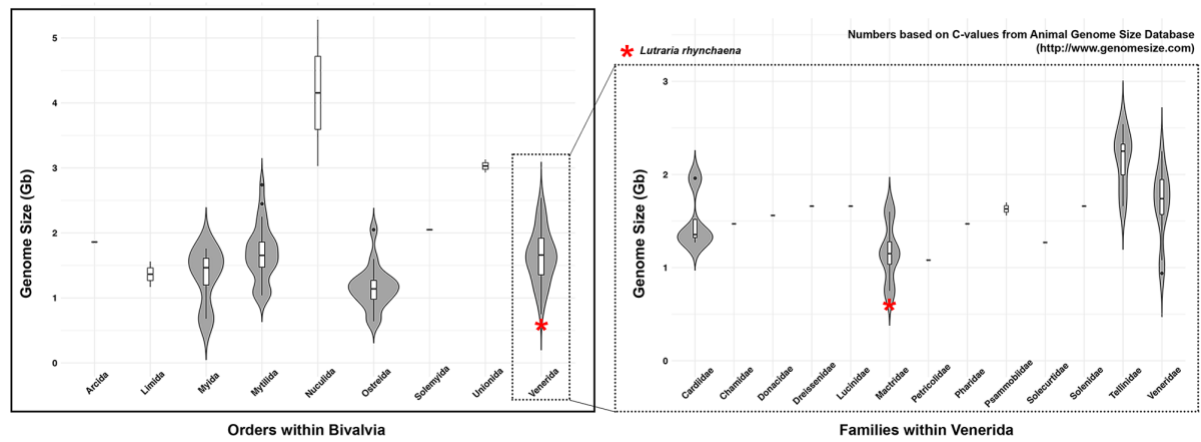

Supplement: Data Sheet 5 — Distribution of genome sizes of bivalve species. [file DataSheet_5.pdf]
